# Motor imagery: lessons learned in movement science might be applicable for spaceflight

**DOI:** 10.3389/fnsys.2015.00075

**Published:** 2015-05-18

**Authors:** Otmar Bock, Nadja Schott, Charalambos Papaxanthis

**Affiliations:** ^1^Institute of Physiology and Anatomy, German Sport UniversityKöln, Germany; ^2^Institute of Sport and Exercise Sciences, University of StuttgartStuttgart, Germany; ^3^Université de Bourgogne, Unité de Formation et de Recherche en Sciences et Techniques des Activités Physiques et SportivesDijon, France; ^4^Institut National de la Santé et de Recherche Médicale (INSERM), UMR U1093, Cognition, Action, et Plasticité Sensorimotrice (CAPS)Dijon, France

**Keywords:** countermeasures, motor performance, mental practice, spaceflight

## Abstract

Before participating in a space mission, astronauts undergo parabolic-flight and underwater training to facilitate their subsequent adaptation to weightlessness. Unfortunately, similar training methods can’t be used to prepare re-adaptation to planetary gravity. Here, we propose a quick, simple and inexpensive approach that could be used to prepare astronauts both for the absence and for the renewed presence of gravity. This approach is based on motor imagery (MI), a process in which actions are produced in working memory without any overt output. Training protocols based on MI have repeatedly been shown to modify brain circuitry and to improve motor performance in healthy young adults, healthy seniors and stroke victims, and are routinely used to optimize performance of elite athletes. We propose to use similar protocols preflight, to prepare for weightlessness, and late inflight, to prepare for landing.

When humans are exposed to weightlessness during spaceflight, they typically exhibit sensorimotor deficits such as spatial disorientation, postural instability and reduced manual dexterity; the symptoms gradually subside over a period of weeks to months, but often re-appear after return to Earth (Clément and Ngo-Anh, 2013). Since these sensorimotor deficits are unpleasant, degrade task performance and increase the risk of accidents, astronauts’ preflight training includes activities during parabolic flight and water immersion, where they experience weightlessness and can pre-adapt to it. Unfortunately, it is difficult to implement similar training regimes to prepare astronauts for their return to Earth, due to the limited availability of equipment and crew time. Here, we propose an approach for a pre-landing training, which requires little time and instrumentation, and which is already used with success in sports. Like astronauts, high-performance athletes face a limited availability of training equipment and a shortage of time, and an established approach to overcome these constraints is the use of motor imagery.

Motor imagery (MI) is a widely used experimental paradigm for the study of action planning and representation (Jeannerod, 1994). MI is described as an active cognitive process during which a specific action is internally reproduced in working memory, from a first-person perspective, without any overt motor output (Decety and Grèzes, 1999). It typically includes multiple sensory modalities, e.g., a person might mentally visualize her arm to move a ball, mentally feel her muscles to contract, and mentally hear the ball’s “thump” against the ground (Weinberg, 2008). Compared to executed movements, imagined movements have similar temporal structure, accuracy and vividness (Guillot and Collet, 2005; Papaxanthis et al., 2012; Schott, 2012), activate similar brain regions in the motor and premotor cortices, the posterior parietal cortex, the basal ganglia and the cerebellum (Jeannerod, 2001; Munzert and Zentgraf, 2009), and are associated with similar autonomic responses (Decety et al., 1991; Mulder et al., 2005).

When motor imagery is scheduled repeatedly rather than just once, for the purposes of training or therapy, it is usually referred to as mental practice (MP; Driskell et al., 1994). It has been documented that MP can improve several aspects of motor performance such as muscle strength (see Yue and Cole, 1992; Ranganathan et al., 2004), movement execution and accuracy (Pascual-Leone et al., 1995; Yágüez et al., 1998; Gentili et al., 2004, 2010), and movement quality (Vogt, 1995). These changes of performance are paralleled by changes of the underlying neuronal circuitry. In a seminal study, Pascual-Leone et al. (1995) used transcranial magnetic stimulation (TMS) to investigate the cortical representation of the hand before and after a 5-day practice of a five-finger exercise on the piano, performed physically in one group and by mental practice in another group. Both groups improved their performance in terms of accuracy and temporal consistency, and the cortical representation of long finger flexor and extensor muscles in contralateral M1 expanded similarly in both groups. Another study (Clark et al., 2014) reported that 4 weeks of wrist-hand immobilization in a control group severely reduced muscle force, impaired voluntary activation and prolonged corticospinal inhibition as assessed by TMS, while the same immobilization in an MP group that practiced 5 days per week reduced muscle force only about half as much, and completely prevented the prolongation of corticospinal inhibition. Similar effects were observed in a group of elderly patients with distal radius fracture (Schott et al., 2013a). Here, the MP group received a 6 weeks regimen consisting of 45 min training every weekday. A significant increase in grip strength and range of motion was observed compared to the control group. Lacourse et al. (2005) observed that 1 week of hand-movement training increases the hemodynamic response of the primary and secondary motor cortex and of the cerebellum by a comparable amount in a physical-practice and in a mental-practice group. Thus taken together, benefits of PM have been reported both at the behavioral and ant the neuronal level for healthy subjects as well as young and elderly patients.

It might seem surprising that MP enhances motor performance even though it provides no sensory feedback from body motion and the environment. The concept of internal models offers a theoretical explanation of this phenomenon. It is thought that neural mechanisms within our sensorimotor system use the currently issued motor commands to simulate the upcoming movement and its sensory consequences; the output of these so-called forward internal models (Kawato et al., 1987) is normally combined with—noisy and delayed—sensory feedback to provide accurate and precise movement estimations (Wolpert et al., 1995). Imagery of movements provides no sensory feedback, but the output of forward internal models is still available for movement estimation (Miall and Wolpert, 1996; Wolpert and Flanagan, 2001). During physical practice, movement estimations serve to refine future motor commands by generating an internal training signal that modifies plastic neural processes (Wolpert et al., 1995; Kawato, 1999; Desmurget and Grafton, 2000) and the same may hold during MP as well, only that the training signal may be less accurate and precise due to the lack of sensory feedback.

Studies on motor imagery in sports can be subdivided into three broad categories. One of them administers a single session of motor imagery along with relaxation techniques prior to a competition; this can enhance athletes’ subsequent performance, probably by adjusting their arousal level and attention focus, and by reducing their anxiety (Hinshaw, 1991; Weinberg, 2008). The second category administers repeated sessions of motor imagery (i.e., MP) as well as of physical exercise; it has been shown that MP enhances progress beyond that achieved by physical practice alone, possibly by serving as a catalyst or as a genuine training stimulus (Taktek, 2004; Weinberg, 2008; Malouin et al., 2013; Vogt et al., 2013). The third category of sports-related research deals with pure mental practice; it reports that MP is efficient, albeit less efficient than pure physical practice (Hinshaw, 1991; Driskell et al., 1994; Schott et al., 2013b). This category is in use for more than 60 years (Twining, 1949); it produced training benefits on tasks ranging from muscle strength building (Wright and Smith, 2009) to gymnastic jumps (Smith et al., 2007), ball games (Wakefield and Smith, 2009; Ramsey et al., 2010) and ski slalom (Callow et al., 2006). The three categories of sports-related imagery research are not equally relevant for pre-landing countermeasures. The first category is less critical since astronauts’ mental preparation for re-entry is typically well developed. The second category is unsuitable for inflight use since imagery training can’t be combined with physical training under terrestrial conditions.^1^ The third category, however, could form a useful approach for pre-landing countermeasures.

MP has been used with success not only in sports, but also in a range of other disciplines. It has been applied to train surgical skills (Immenroth et al., 2007; Cocks et al., 2014), enhance music performance (Keller, 2012), improve balance in the elderly (Hamel and Lajoie, 2005), and support orthopedic and neurological rehabilitation (Jackson et al., 2001; Schott and Korbus, 2014). Several frameworks have been presented in the literature to define the key components of successful MP (for an overview see Guillot and Collet, 2008; Schott et al., 2013a); among them, the PETTLEP model (Holmes and Collins, 2001) emerged as the most promising one. This framework is elaborated by the Functional Equivalence Model, which states that motor imagery should resemble the actual task in seven critical aspects, summarized by the acronym PETTLEP (Holmes and Collins, 2001). This acronym stands for *Physical* (body should assume the same physical postures as in the actual task, rather than being relaxed as in many earlier studies), *Environment* (imagery should include the same architecture and furnishing as the actual task), *Task* (same body and eye movements, muscle tensions, heart rate, dry mouth etc. as in actual task), *Timing* (not in slow motion as in some earlier studies), *Learning* (imagery should be modified as practice progresses), *Emotion* (same excitement and arousal as with actual task, but avoid negative feelings) and *Perspective* (the 1st person perspective is more efficient in most tasks, but there are exceptions).

It became standard practice within the last decade to guide subjects’ motor imagery by scripts. This approach has been formalized in detail by Eberspächer and colleagues (Immenroth et al., 2007). First, experimenter and trainees observe and discuss the desired motor activity and its sensory, autonomic and emotional concomitants. The outcome is then fixed in written form to enhance precision. The activity is subsequently broken down into key components or “nodal points”, and each nodal point obtains a verbal label such that calling it up is facilitated. In a final step, the key characteristics are marked symbolically, i.e., they are renamed as individual short formulae (e.g., up—centered or pull—release for dorsal flexion). The aim is the compression of the information (cognitive chunking) of body postures and movements. The image should thus be approximated to the dynamics and temporal duration of the real movement. In each session, the trainee reads or hears the script and then engages in the pertinent imagery. Due to findings of Yao et al. (2013), that mental imagery is most effective with a kinesthetic focus, the training process should aim at that modality.

A series of experiments by Smith and colleagues (Smith et al., 2007) provides good examples for successful motor imagery training based on PETTLEP and scripting. The experimenter formulated with each trainee an individualized script which included descriptions of visual and kinesthetic inputs, motor outcomes, smells and emotions; these scripts were modified as practice progressed, to satisfy the Learning principle of PETTLEP. Imagery sessions were scheduled in the trainees’ normal sports facility, with participants wearing their normal sports clothes. PETLEP training was not as effective as physical training, but was more effective than standard imagery in seated and relaxed subjects.

Given its success on Earth, MP based on PETTLEP and scripting could also be useful for pre-adapting astronauts to the terrestrial environment while they are still in orbit. We suggest a training regime where individualized scripts are established and refined before launch, and are used for MP during the final days before landing.

The first phase would take place on Earth, in the astronauts’ familiar environment. They would wear their regular outfit and perform their typical daily activities such as standing up, walking with and without a load, sitting down, manually retrieving, handling and replacing of objects, etc. Astronauts would then engage in mental practice of these activities, using the scripts, to provide the individually relevant functional, sensory and emotional aspects of the reference movements, their dynamics and their timing. In a next step, astronauts would elaborate the key characteristics of the activities in form of scripts. To this end, they assume and maintain the starting posture of the respective physical movement, execute the activities with eyes open, with eyes closed (to enhance kinesthetic perception, see Yao et al., 2013) and mentally, and then report their sensations, observations and emotions to the instructor. Once the scripts are formulated in several iterative steps, the key movement characteristics are marked symbolically, e.g., the sequence “stand up—walk—turn—walk—sit down” could be labeled “TUG”, as in the timed-up-and-go test. To assess training progress, astronauts would signal the onset and completion of each imagined sub-movement to the observer by slightly lifting their hand; the systematic and variable errors between the timing of imagined and physical movements could then serve as markers of progress.

The second phase would start several days before landing, and consist of multiple sessions in which astronauts listen to the short-formed scripts and then mentally imagine the pertinent sensations, observations and emotions. They would not engage in those activities physically, since in the absence of gravity, this would defeat the purposes of training. Again, astronauts would use hand signals to allow judgments of their progress.

We expect that in analogy to MP in sports, MP before landing will improve astronauts’ sensorimotor performance upon return to Earth, and will thus serve as an effective countermeasure. For example, practice of the above “TUG” script could enhance astronauts’ ability for standing up and walking without assistance after landing, and the benefit could generalize to other postural tasks as well.

Mental practice could be applied to prepare astronauts not only for the sudden onset of gravity upon landing, but also to the sudden absence of gravity after launch. Presently, this preparation is limited to parabolic flights—which offer only brief periods of weightlessness that alternate with periods of hypergravity, and to water immersion—which introduces confounding factors such as viscosity, slight nitrogen narcosis (Dalecki et al., 2012) and persistence of normal vestibular inputs. Mental practice could offer an expedient alternative or supplement to those established methods. Likewise, mental practice could be applied to prepare astronauts for the landing on and the launching from celestial bodies other than the Earth. Finally, MP could be used as countermeasure for decrements not only of motor skill, but also of muscular force: particularly MP with kinesthetic imagery was found to counteract the loss of muscle strength following immobilization (Meugnot et al., 2015) without inducing muscular fatigue (Rozand et al., 2014); the effects of MP on muscular force are not much smaller than those of physical practice (Reiser et al., 2011).

## Conflict of Interest Statement

The authors declare that the research was conducted in the absence of any commercial or financial relationships that could be construed as a potential conflict of interest.

## Footnotes

^1^Although payload such as treadmills equipped with bungee cords is available aboard the ISS, such hardware simulates some but not other impacts of terrestrial gravity. This hardware is valuable for the training of strength and endurance, but it would be inadequate for the training of sensorimotor coordination where it even might produce negative transfer.
